# Supplementary material for: MdMYB66 Is Associated with Anthocyanin Biosynthesis via the Activation of the MdF3H Promoter in the Fruit Skin of an Apple Bud Mutant
Source: Int J Mol Sci. 2023 Nov 28;24(23):16871. doi: 10.3390/ijms242316871 (PMC10706036; doi:10.3390/ijms242316871)
Supplement: Supplementary file 1 [file ijms-24-16871-s001.zip › Supplementary Table S1.pdf]

Supplementary Table S1. The list of 25 anthocyanins detected between OS and RM.

Metabolites	Q1 (Da)	Q3 (Da)	RT	OS-S1	RM-S1	OS-S2	RM-S2
Cyanidin-3-O-glucoside	449.1	287.1	10.22	0.5322±0.0596	3.9357±0.3235	3.9775±0.0779	11.3385±0.2051
Cyanidin-3-O-(6-O-p-coumaroyl)-glucoside	595.1	287.1	5.39	0.0318±0.0043	0	0	0
Cyanidin-3-(6-O-p-caffeoyl)-glucoside	611.1	287.1	5.59	0	0.0086±0.0012	0	0.0284±0.0024
Cyanidin-3-O-sophoroside	611.2	287.1	6.84	0	0	0.0350±0.0015	0.17634±0.0200
Cyanidin-3-O-rutinoside	595.1	287.1	7.41	0.0878±0.0216	0.0640±0.0110	0.0649±0.0069	0.1377±0.0098
Cyanidin-3-O-arabinoside	419.1	287.1	7.52	0.9748±0.0258	9.1665±0.5586	12.1915±0.3732	30.2992±0.7975
Cyanidin-3-O-galactoside	449.1	287.1	7.85	144.2347±1.3855	807.8117±23.352	878.8717±6.7694	1514.9780±19.2482
Cyanidin-3-O-xyloside	419.1	287.1	9.64	1.6482±0.0582	20.0588±0.8656	28.4936±0.8642	74.5131±2.4180
Delphinidin-3-O-(6-O-acetyl)-glucoside	507.1	303.1	5.97	0.5171±0.0063	0.4704±0.0165	0.1366±0.0126	0.1653±0.0066
Delphinidin-3-O-glucoside	465.1	303.1	6.30	0	0	0.0210±0.0023	0.0765±0.0027
Pelargonidin-3-O-galactoside	433.2	271.1	6.22	0	0	0.3016±0.0099	1.1281±0.0532
Peonidin-3-O-rutinoside	609.5	301.1	6.71	0	0	0.0066±0.0160	0.0190±0.0005
Peonidin-3-O-glucoside	463.3	301.1	8.96	0.0028±0.0004	0.0214±0.0012	0.0939±0.0094	0.5044±0.0318
Peonidin-3-O-arabinoside	433.2	301.1	9.38	0.0545±0.0013	0.15317±0.0120	0.5718±0.0160	2.4680±0.0797
Procyanidin C1	867.2	579.1	3.54	663.6436±15.2701	656.9184±62.4235	262.3470±25.8185	174.3044±23.8325
Procyanidin B3	579.1	427.1	3.88	51.9806±2.1106	19.7772±0.0250	2.5293±0.0613	2.5612±0.0529
Procyanidin B2	579.1	427.1	5.54	673.0564±10.5384	632.9630±8.5054	658.0235±9.6711	624.6809±4.3347
Procyanidin B1	579.1	427.1	6.79	258.9216±7.9578	129.6271±0.7898	69.1411±1.6243	52.9580±2.3869
Procyanidin A2	577.3	425.1	7.15	0	0	0.8356±0.0405	2.7956±0.2005
Procyanidin A1	577.1	425.1	9.21	10.4048±0.7148	11.0927±0.5164	0	0
Rutin	611.2	303.1	10.85	24.1444±0.7141	19.5763±0.8004	7.1411±0.6231	7.9661±0.4683
Afzelin	433.1	287.1	11.34	6.1795±0.2342	5.8983±0.1622	1.7338±0.0987	1.5466±0.0408
Kaempferol-3-O-rutinoside	595.2	287.1	11.35	0.4761±0.0571	1.0031±0.0778	0.3043±0.0245	1.6776±0.1598

Supplementary Table S1 (continued)

Quercetin-3-O-glucoside	465.1	303.1	11.46	64.5471±2.5362	56.7529±0.9340	45.7790±2.7298	40.1944±1.8418
Naringenin	273	153.1	12.51	0.0372±0.0060	0.0480±0.0043	0	0
