# Supplementary material for: MdMYB66 Is Associated with Anthocyanin Biosynthesis via the Activation of the MdF3H Promoter in the Fruit Skin of an Apple Bud Mutant
Source: Int J Mol Sci. 2023 Nov 28;24(23):16871. doi: 10.3390/ijms242316871 (PMC10706036; doi:10.3390/ijms242316871)
Supplement: Supplementary file 1 [file ijms-24-16871-s001.zip › Supplementary Table S2.pdf]

Supplementary Table S2. Identification of the differentially accumulated anthocyanins (DAAs) in OS and RM.

Stage	Metabolite	VIP	FC(RM/OS)	P-value	Type
20DAF	Cyanidin-3-O-(6-O-p-coumaroyl)-glucoside	1.0094	0.0104	7.39E-06	down
	Cyanidin-3-O-arabinoside	1.0091	2.6253	8.08E-06	up
	Cyanidin-3-O-galactoside	1.0070	1.5640	1.29E-05	up
	Cyanidin-3-O-xyloside	1.0099	3.3998	5.44E-05	up
	Delphinidin-3-O-(6-O-acetyl)-glucoside	1.0093	0.2539	5.54E-05	down
	Naringenin-7-O-glucoside	1.0087	0.3391	0.0001	down
	Procyanidin A1	1.0082	0.2983	0.0012	down
	Procyanidin B1	1.0098	0.1399	0.0013	down
	Procyanidin B2	1.0092	0.2626	0.0013	down
	Procyanidin B3	1.0099	0.1063	0.0040	down
	Procyanidin C1	1.0077	0.2763	0.0061	down
	Quercetin-3-O-glucoside	1.0101	0.2458	0.0080	down
	Rutin	1.0089	0.2264	0.0137	down
	Afzelin	1.0099	0.2667	6.48E-08	down
130DAF	Cyanidin-3-(6-O-p-caffeoyl)-glucoside	1.0217	5.9961	0.0002	up
	Cyanidin-3-O-arabinoside	1.0174	1.5736	0.0022	up
	Cyanidin-3-O-glucoside	1.0187	1.8059	1.36E-05	up
	Cyanidin-3-O-sophoroside	1.0170	3.1783	0.0007	up
	Cyanidin-3-O-xyloside	1.0186	1.6556	0.0017	up
	Delphinidin-3-O-glucoside	1.0049	2.3071	0.0009	up
	Kaempferol-3-O-rutinoside	1.0160	3.4885	5.38E-05	up
	Naringenin-7-O-glucoside	1.0207	0.7982	0.0008	down
	Pelargonidin-3-O-galactoside	1.0209	2.3677	5.52E-05	up
	Peonidin-3-O-arabinoside	1.0215	2.7323	0.0004	up
	Peonidin-3-O-glucoside	1.0117	3.4079	0.0001	up
	Peonidin-3-O-rutinoside	1.0038	1.8318	0.0017	up
	Procyanidin A2	1.0180	2.1171	2.64E-05	up
	Procyanidin B1	1.0159	0.4856	0.0012	down
	Procyanidin B2	1.0197	0.6014	5.56E-05	down
	Procyanidin B3	1.0127	0.6416	0.0002	down
	Quercetin-3-O-glucoside	1.0006	0.5566	0.0006	down
	Afzelin	1.0057	0.5652	0.0004	down
