# Supplementary material for: MdMYB66 Is Associated with Anthocyanin Biosynthesis via the Activation of the MdF3H Promoter in the Fruit Skin of an Apple Bud Mutant
Source: Int J Mol Sci. 2023 Nov 28;24(23):16871. doi: 10.3390/ijms242316871 (PMC10706036; doi:10.3390/ijms242316871)
Supplement: Supplementary file 1 [file ijms-24-16871-s001.zip › Supplementary Table S3.pdf]

Supplementary Table S3.1 Sequence of primers used for qRT-PCR analysis.

Gene ID	Gene name	Forward primer (5' to 3')	Reverse primer (5' to 3')
MD10G1097200	CAD	GGCTGCTGACTCCTTGGATTACATC	GCCGTCAAGTTTCAACAAAGAGAGG
MD15G1246200	F3H	CACCGTTCAACCAGTGGAAGGAG	GCTGGGTTCTGGAATGTGGCTATG
MD01G1236300	4CL2	AAGGAACCGATGCCAACCAAGTC	GCCGAGTGAGAGACCAGTTTCAAG
MD17G1229400	4CL1	TCCACTCCTACTGCCTCCACAAC	CATCGGCGAAGGTGTATATGTCTCC
MD01G1237600	CCR	ATGGCAGAGGCAGAGGTGAGAG	GAGGACGCTGACGACGCATTAC
MD12G1184600	POD	CTTGTGCTGACATCTTAACCGTTGC	GCTTGCTGTGGTGGAGTCTCTTC
MD15G1377800	bHLH96	TCAATCTTGCCCATCAGGACAACAC	ACAATGTGCGTCATCCTCTGGTTC
MD09G1278600	MYB113	ATCGACTGTCGCCAAGATCATGC	TGATTCCAAAGGTCCGTGCTAAAGG
MD15G1050300	MYB114	TGGTTCCAGTTAGCAGTCGAAGTTC	GCCTCGGACCGTGGATTTCTATAAC
MD14G1189300	MYB1R1	ACTTGAACTCAACGACGGACACATC	TGTCTGGAAAGCCGAATGCCTTG
MD15G1051400	MYB6	GAAGTACCGAAGAGTTTCCGAGGAC	TGTCGTCATCATCGCAGCCAAAG
MD04G1185100	PIF3	GCCAGCCAATTCTAGTGAGATCCAG	CCGCTTAGCAGTACATGGTTCCTC
MD09G1106400	CPC	CCACTCCACTTCTGACGACAACTC	TCCCAGCAATCAGAGACCACCTC
MD14G1181000	WER	CTAAGTCCCAGCGTGAAGAGAAGTG	TGTTGCCAATGAGGTTATGGAGTCG
MD14G1197600	PRE6	GGTATCGGCGTCGAGCATTTGG	TTCTTTGGCTCAGATCTTCCACCTC
MD05G1074600	HHO6	TGGCTTGACGAATGACGAAGTGAAG	CCCCAAACCAACAGATTGCTTTTCC
EB146750	MdGADPH	TTCTCGTTGAGGGCTATTCCA	CCACAGACTTCATCGGTGACA

Supplementary Table S3.2 The Primers sequence for gene amplification and plasmid construction.

Gene name	Forward primer (5' to 3') and reverse primer (5' to 3')
MdMYB66-GFP	F:gagctcggtacccggggatccATGGAAGGTGGGAATGAGTCTAAG
	R:ggtgtcgactctagaggatccCAAATAATTATCAAATTGAAAAGGGTAG
MdMYB66(pGADT7)	F:gtgggcatcgatacgggatccttATGGAAGGTGGGAATGAGTCTAAG
	R:cagctcgagctcgatggatccTTACAAATAATTATCAAATTGAAAAGGG
MdMYB66(pHIS2)	F:gactcactatagggcgaattcTCGTCTCAGTGAGTATTTGATCAAAG
	R:gcgtgagctccccgggaattcTTTTGTGTTTGTCTTGGTGAGGC
MdMYB66(pGBKT7)	F aggccgaattcccggggatccATGGAAGGTGGGAATGAGTCTAAG
	R ccgctgcaggtcgacggatccTTACAAATAATTATCAAATTGAAAAGGG
MdMYB66(pGreenII 62-SK)	F:aggacagcccaagctgagctcATGGAAGGTGGGAATGAGTCTAAG
	R:gataagcttgatatcgaattcTTACAAATAATTATCAAATTGAAAAGGG
MdF3H(pGreenII 0800-LUC)	F:ggtaccgggccccccctcgagTCGTCTCAGTGAGTATTTGATCAAAG
	R:caggaattcgatatcaagcttTTTTGTGTTTGTCTTGGTGAGGC

Supplementary Table S3.3 The primer sequence of transgenic material qRT-PCR.

Gene ID	Gene name	Forward primer (5' to 3')	Reverse primer (5' to 3')
MD04G1003300	MdCHS	ATCACCAACAGCGAGCACAAG	CTTCCACAACCACCATGTCCTG
MD07G1186300	MdCHI	TTCCACCGTCCGTCAAACCT	CGGCGTTATCCTCCAAGTACAC
MD15G1024100	MdDFR	ATCGGCTCTTGGCTCGTCAT	CCTTCGGCAAGTCCAACAGATG
MD15G1246200	MdF3H	ATGGCTCCTCCTGCTACTACG	GGCAAGCGAGATGATCGGAAT
MD06G1071600	MdANS	GAGGAAGTTCGCAAGGCTCAA	CTTGTGCTCGCTGTTGGTGAT
MD01G1234400	MdUFGT	GCTCCATCCTCCGTCGTGTA	GCCTCCGCTATTGCCATCTG
	MdMDH	CGTGATTGGGTACTTGGAAC	TGGCAAGTGACTGGGAATGA
MD14G1181000	MdMYB66	CCAGCGTGAAGAGAAGTGACTT	AAGACCACCTGTTGCCAATGAG
	Hyp	ATGAAAAAGCCTGAACTCACCG	CTATTTCTTTGCCCTCGGACGA
