# Supplementary figures and images for: MdMYB66 Is Associated with Anthocyanin Biosynthesis via the Activation of the MdF3H Promoter in the Fruit Skin of an Apple Bud Mutant
Source: Int J Mol Sci. 2023 Nov 28;24(23):16871. doi: 10.3390/ijms242316871 (PMC10706036; doi:10.3390/ijms242316871)

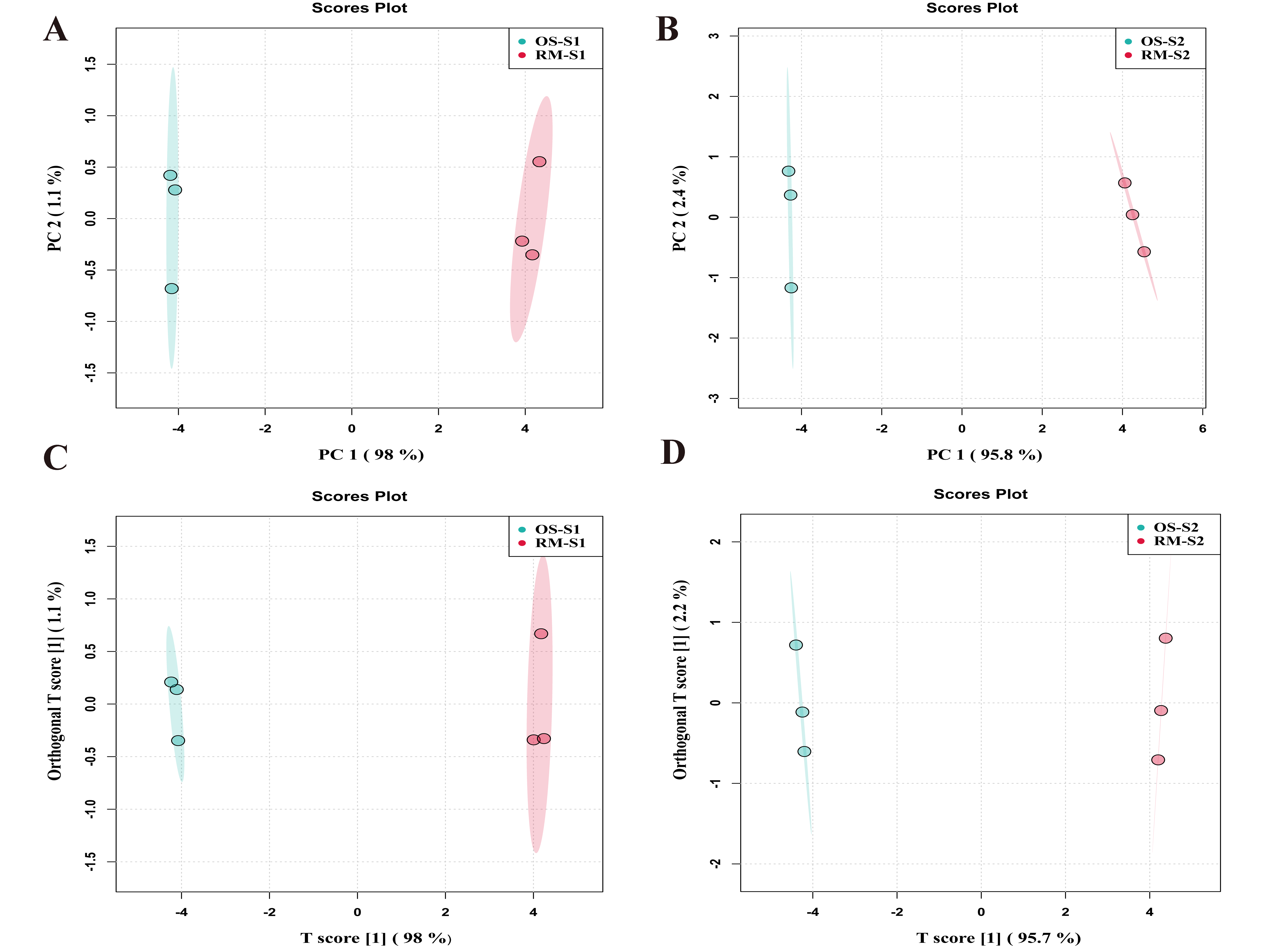

Supplement: Supplementary file 1 [file ijms-24-16871-s001.zip › Figure S1.tif]

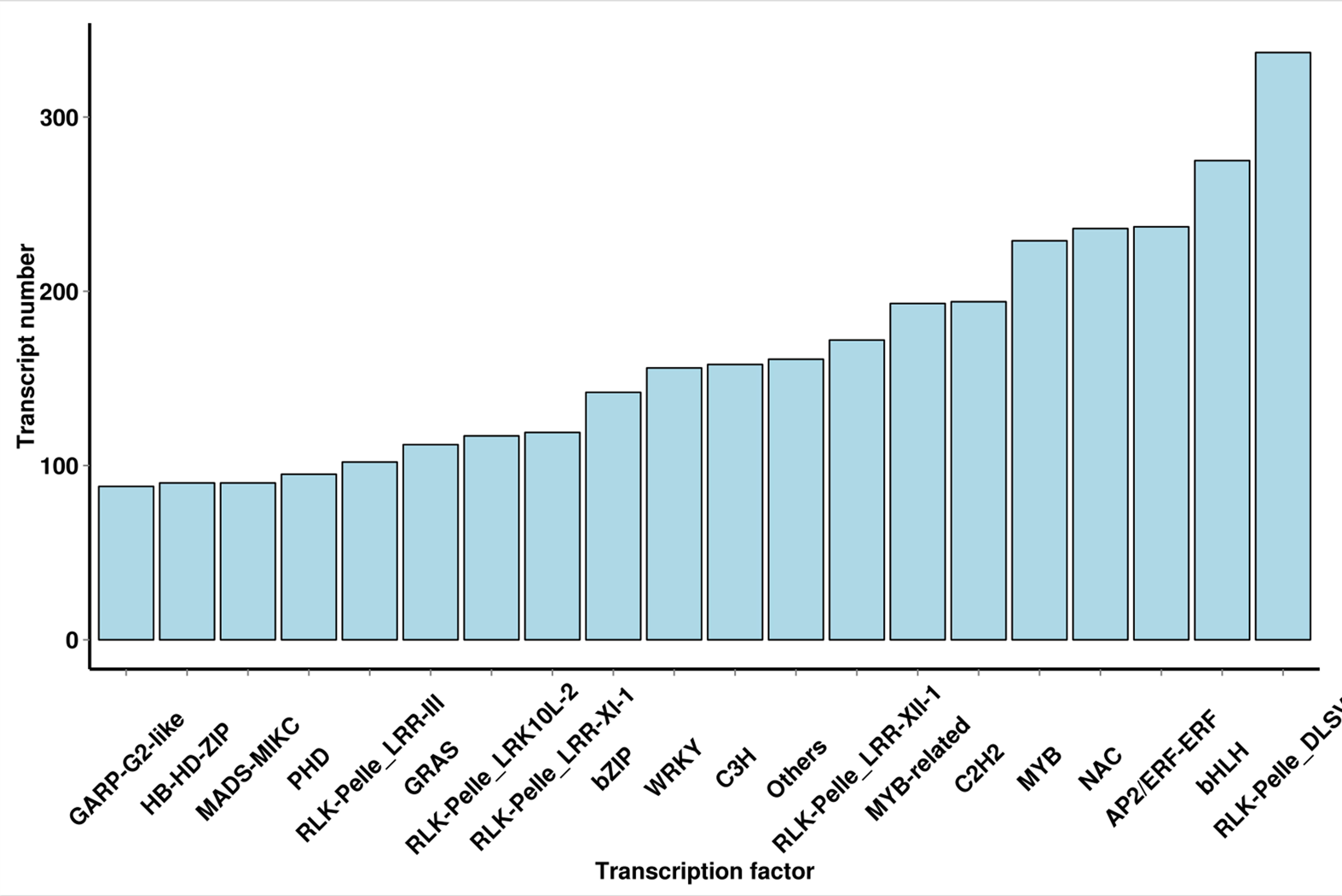

Supplement: Supplementary file 1 [file ijms-24-16871-s001.zip › Figure S2.tif]
